# Endometriosis in an ectopic kidney: a rare case report and literature review

**DOI:** 10.1186/s12905-023-02343-x

**Published:** 2023-04-28

**Authors:** MengLin Chen, YuanMeng Yu, XinXiang Zhao

**Affiliations:** 1 Department of Radiology, The second Affiliated Hospital of Kunming Medical University, Kunming, Yunnan, 650032 China; 2 Department of MRI, The First People’ s Hospital of Yunnan Province, The Affiliated Hospital of Kunming University of Science and Technology, Kunming, Yunnan, 650032 China

**Keywords:** Endometriosis, Renal endometriosis, Ectopic kidney

## Abstract

**Background:**

Endometriosis mainly occurs in female pelvic organs. Endometriosis in the kidney is extremely rare.

**Case presentation:**

We herein describe a case of a 19-year-old girl with occasional mild abdominal pain associated with an ectopic left kidney. SPECT-CT showed no abnormal radioactive distribution in the left pelvis, suggesting loss of function of the ectopic kidney. Laparoscopic left ectopic kidney resection was subsequently performed. Histopathology revealed endometriosis of the ectopic left kidney.

**Conclusions:**

In female patients with clinical manifestations of abdominal pain and gross hematuria, the possibility of renal endometriosis should be considered.

## Background

Endometriosis, the presence of endometrial glands and stroma outside the uterine cavity, is the second most common pelvic disorder and the most common cause of pelvic pain in women of reproductive age [1]. Most lesions usually located within the reproductive system, but rarely occurs outside the pelvis, especially in the kidney [2].

Endometriosis in the ectopic kidney is very rare and has not been reported in the literature yet, to the best of our knowledge. Herein, we report our experiences with regard to the clinical and imaging manifestations of endometriosis in an ectopic kidney to raise awareness of this rare disease.

## Case presentation

A 19-year-old girl was admitted to our hospital for an ectopic left kidney during physical examination. She noted occasional mild peri-abdominal pain. Otherwise, she was healthy and physical examinations were unremarkable.

### Radiographic findings

**Fig. 1:**
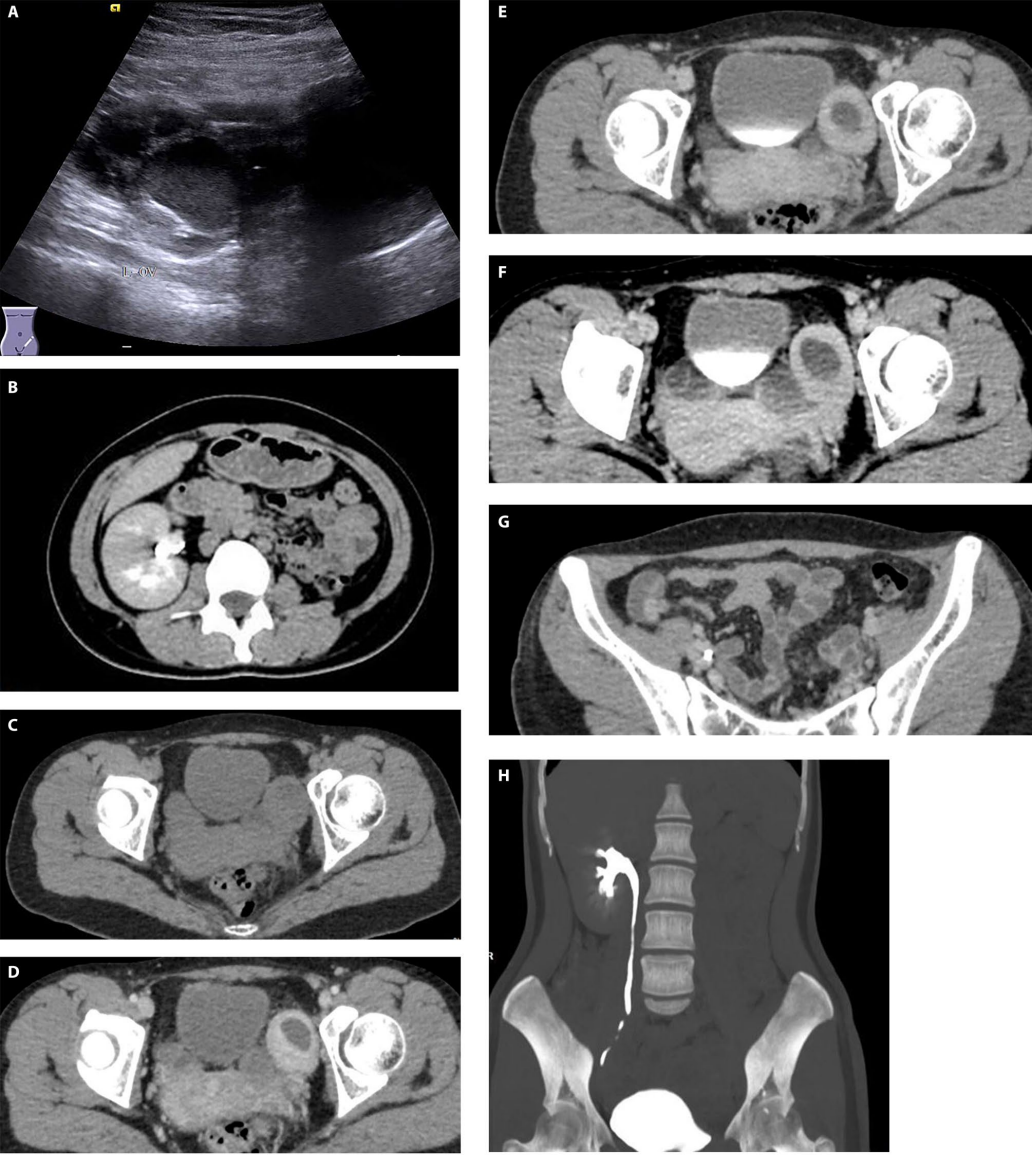
(A):Ultrasound of the left side of the pelvis showed a substantial inhomogeneous echogenic structure with clear boundary, and an irregular shape. (B,H):Computed tomography (CT) showed no indication of kidney in the left renal area. CT showed a hypodense mass of 1.6 × 2.2 cm in the left ectopic kidney. (C) :Unenhanced images. (D): Renal cortical phase. (E) :Renal corticomedullary phase. (F):Renal excretory phase. (G): the ureter can be seen as dilated and hydrous in the left ectopic kidney

An ultrasound of the urinary system was performed first that showed absence of kidney in the left renal region. However, on the left side of the pelvis, a substantial inhomogeneous echogenic structure measuring about 7.3 × 4.6 cm with a clear boundary and an irregular shape was detected. The left ureter is not shown clearly (Fig. 1A).

Subsequent abdominal computed tomography (CT) indicated absence of kidney in the left renal area (Fig. 1B, H). A large and heterogeneous hypodense mass was observed in the pelvic cavity. After contrast injection, the solid areas showed mild enhancement, and the cystic components remained unenhanced (Fig. 1C-F). In addition, the ureter can be seen as to be dilated and hydrous (Fig. 1G).

**Fig. 2:**
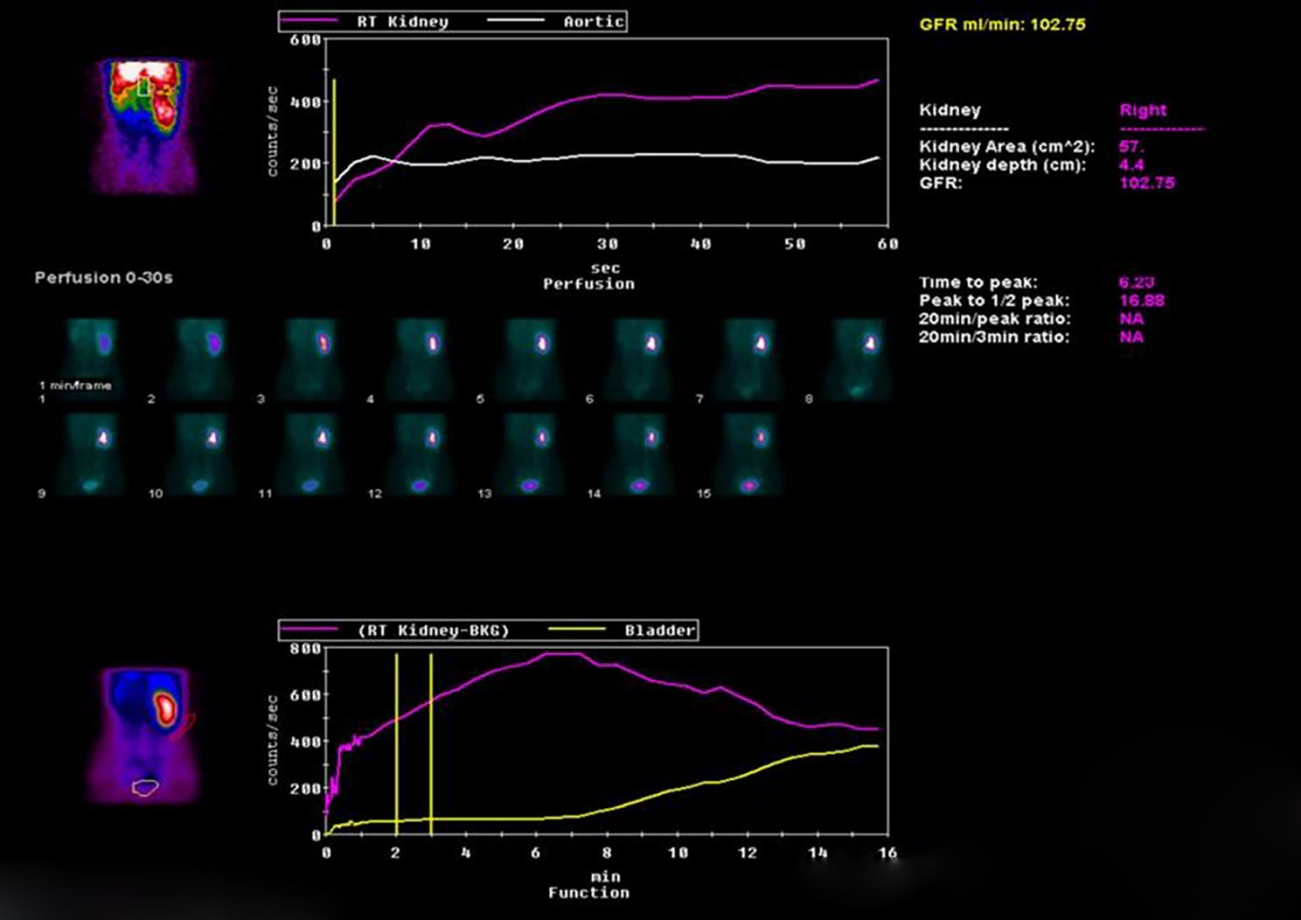
99mTc-DTPA renal scan showed the left kidney was not visualized, and the right kidney was clearly visualized

The patient also underwent 99mTc-DTPA renal scan (Fig. 2). Immediately after intravenous “bolus” injection of 99mTc-DTPA, posterior biphasic renal artery imaging was collected and glomerular filtration rate was measured. The left kidney was not visualized, and the right kidney was clearly visualized on renal artery perfusion phase. On the renal cortical function phase, no abnormal radioactive distribution was found in the left kidney. Reversely, the location, size and shape of the right kidney were normal. The radioactivity of the right kidney was evenly distributed, and there was no abnormal radioactive concentration or defect. The radioactivity of the right kidney reached a peak at 6 min, and then gradually decreased with time; the bladder was gradually developed at about 7 min on clearance phase. Nephrogram curve showed that no obvious abnormality of curve in the right kidney was found.

### Surgical procedure and pathological findings

Laparoscopic left ectopic kidney resection was subsequently performed at our hospital. After entering the pelvic cavity, the hydrous kidney and dilated ureter are located on the left side of the pelvic cavity and adhered to the surrounding tissue. The uterus is located at the center of pelvis. Ovaries and fallopian tubes are seen on the right side of the pelvic cavity. No obvious left fallopian tubes and ovaries are observed. Finally, it was considered that the left ectopic kidney of the pelvic cavity combined with unilateral accessory (right side). Resected specimens were examined by family members and then sent for pathological examination.

**Fig. 3:**
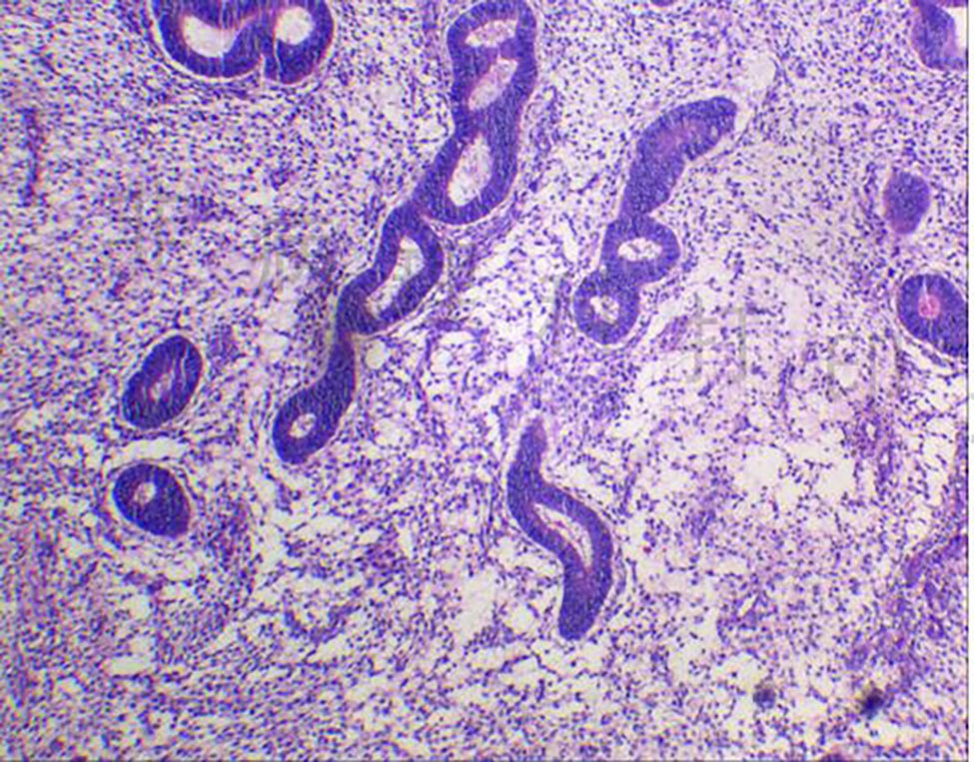
The microscopic pathology proved the diagnosis of renal endometrios was characterized by endometrial glands [hematoxylin and eosin stain (H and E), × 200 magnification]

The size of the resected mass was approximately 6.5 × 4.5 × 3.5 cm, and the cut surface of the mass was several capsular spaces containing brown fluid. Microscopic examination confirmed the diagnosis of ectopic renal endometriosis characterized by endometrial glands and tubal component (Fig. 3).

## Materials and methods

Renal endometriosis was first reported by Marshall in 1943 [4]. It is very rare in the past 30 years, and only 17 cases of renal endometriosis have been reported [5–20]. To our knowledge, this is the first report of endometriosis within ectopic kidney.

### Literature review

A total of 17 patients (age range, 23–53 years) with pathologically proven renal endometriosis from 1970 to 2021 were included in our retrospective review. Patients were identified in PubMed using the keywords: “renal endometriosis”. Clinical features, endometrial history, tumor size, diagnosis method, treatment, and outcome were recorded.

### Statistical analysis

Continuous variables were expressed as means ± standard deviation, and categorical variables as number (percentage).

## Results

Demographic and clinical findings of the renal endometriosis reported in the previous literature are summarized in Table 1.

**Table 1:** Case reports of renal endometriosis

Reporting year	Location	Age	Clinical features	Positive history for endometriosis diagnosis	Size(cm)	Diagnosis method	Treatment	Outcome	Reference
1970	Left kidney	49	Low back pain and gross hematuria	No	1.5 × 1.0	Microscopic examination	No	Die	[32]
1977	Right kidney	23	Tender to palpation in the region of the right kidney	Not mentioned	2	Histopathology	Nephrectomy	Recovery	[31]
1980	Upper pole of the right kidney	40	Dull aching pain in the right loin	Not mentioned	2	Histopathology	Nephrectomy	Not mentioned	[30]
1991	Upper pole of the left kidney	25	Macroscopic hematuria and back-pain	Endometriotic cyst of left ovary	4	Ultrasound-guided FNA	GnRha	Effective	[29]
2001	Left kidney	35	Fever and pyuria	Not mentioned	Not mentioned	Histopathology	Nephrectomy	Not mentioned	[28]
2005	Right kidney	40	Pain in the lower abdomen	Endometriotic cyst of right ovary	2	Ultrasound-guided FNA	Not mentioned	Not mentioned	[27]
2006	Right kidney	38	Abdominal pain	Endometriotic cyst of left ovary	1	FNA	GnRha	The lesions regressed slowly	[26]
2008	Left kidney	25	Lower abdominal pain	Not mentioned	25 × 15	Histopathology	Nephrectomy	Recovery	[25]
2009	Lower pole of the left kidney	46	Left lumbar pain and lumbar mass	No	15 × 9.7 × 9.5	Histopathology	The renal capsule was incised and excised after hematoma drainage	Recovery	[24]
2013	Middle and lower of the right kidney	42	Right flank pain and hematuria	No	13.5 × 12 × 12	Histopathology	Nephrectomy, GnRHa	Recovery	[23]
2015	Lower pole of the right kidney	37	Dull pain in the right lower back	Not mentioned	7.5 × 5 × 3.5	Histopathology	Nephrectomy	Not mentioned	[22]
2015	Right kidney	53	Intermittent recurrent right flank pain	Not mentioned	Not mentioned	Histopathology	Nephrectomy after drainage	Recovery	[21]
2017	Upper and lower pole of the left kidney	40	No	Ovarian endometriosis	1.8	Histopathology	No	No clinical changes	[20]
2017	Middle pole of the left kidney	39	No	Ovarian endometriosis	<1.0	Histopathology	No	No clinical changes	[20]
2018	Upper pole of the left kidney	45	Flank pain and intermittent gross hematuria	No	2.8 × 2.6 × 1.7	Histopathology	Partial nephrectomy	Not mentioned	[19]
2020	Right kidney	30	Dull pain in the right lower back	Not mentioned	6.5 × 5.9 × 5.7	Histopathology	Nephrectomy	Not mentioned	[18]
2021	lower pole of the right kidney	48	intermittent gross hematuria	No	1.8 × 1.5 × 1.4	Histopathology	Nephrectomy	Recovery	[5]

Approximately half of the patients (47.1%) were found in the left kidney, 9 patients (52.9%) in the right kidney (Table 2). Abdominal pain was the most frequent presenting symptom (61.9%), followed by gross hematuria (23.8%), other uncommon symptoms (4.8%) (Table 2). The mean age of the patients was 38.5 ± 8.7years (range,23–53 years). The average tumor size was 5.5 ± 6.9 cm (range, 1–25 cm) (no data for 2 patients) in greatest diameter.

**Table 2:** Features characterizing renal endometriosis presentation in literature review

	n(%)
Location	
Left kidney	8(47.1%)
Right kidney	9(52.9%)
Symptoms	
Pain	13(61.9%)
Gross hematuria	5(23.8%)
Others	1(4.8%)
No symptoms	2(9.5%)
Endometrial history	
Ovarian endometriosis	5(29.4%)
No	6(35.3%)
Not mentioned	6(35.3%)
Tumor size(in greatest diameter)	5.5 ± 6.9
Age	38.5 ± 8.7
Diagnosis method	
Histopathology	13(76.5%)
Other	4(23.5%)
Outcome	
Recovery	6(35.3%)
Died	1(5.9%)
No mentioned	6(35.3%)
Others	4(23.5%)

Note:4 cases presented with clinical manifestations of both pain and hematuria. 2 cases no data for size

## Discussion and conclusions

Endometriosis is the second most common pelvic disorder and the most common cause of pelvic pain in women of reproductive age [1]. The main site of the disease is ovary, and the urinary tract involvement is rare, mainly the bladder (80%), followed by ureters (15%) and kidneys (<5%) [21]. To date, only 17 cases of renal endometriosis have been reported, the first case was reported by Marshall in 1943 [3]. To our knowledge, this case is the first report of endometriosis within ectopic kidney.

Several theories of the pathogenesis of endometriosis have been proposed, including embryonic, migratory, and immunologic theories [4–7]. Concretely, embryonic theories indicate that endometriosis results from metaplastic changes of Wolffian, Mullerian, and occasionally peritoneal (celomic) structures. Migratory theories suggest that retrograde menstruation, lymphovascular metastasis, and direct extension allow for transplantation of the endothelial cells into ectopic sites. Immunologic theories suggest that a suboptimal immune response maybe result in ectopic endometrial implantation [6–36]. Simone Laganà et al. summarized recent evidences about the pathogenesis of endometriosis in remote sites and believed the retrograde menstruation of stem/progenitor cells from endometrial niches to the peritoneal cavity and the migration of bone marrow-derived stem cells through peripheral circulation may underlie the development of endometriosis in remote sites (e.g., kidney, nose and so on) [6–10]. In addition, accumulating evidence demonstrates that immune cells, adhesion molecules, extracellular matrix metalloproteinase and pro-inflammatory cytokines activate/alter peritoneal microenvironment, creating the conditions for differentiation, adhesion, proliferation and survival of ectopic endometrial cells [6–9]. For example, a recent study found that the quantity of M1 and M2 macrophages in ovarian endometriomas at different stages of the disease were different and implied that the activity and polarization of macrophages maybe play a key role in development of endometriosis [10]. Some researches proposed new insights on the pathogenesis and pathophysiology of endometriosis from novel perspectives. Such as, Murgia et al. applied a metabolomic strategy to explore metabolic alteration in patients with endometriosis to better understand the pathophysiology of endometriosis [11]. Viganó et al. indicated that small bowel permeability may be associated with the maintenance of low-grade inflammation of endometriosis [12].

Endometriosis is regarded as an estrogen-dependent process, with clinical symptoms of dysmenorrhea, dyspareunia, and profuse bleeding [4]. Nevertheless, the clinical presentation of renal endometriosis is diverse and atypical. The absence of typical clinical manifestations may be due to the fact that the renal endometriosis is confined to the renal cortex without involvement the renal calyces. According to previous reports, most of the clinical manifestations of renal endometriosis are abdominal pain and gross hematuria. Long-term periodic bleeding may lead to the gradual increase of hemorrhagic cysts in the kidney tissue and even invasion into the renal calyces, which can result in ureteral obstructions, and that is the main cause of abdominal pain and hematuria [13]. Although peritoneal superficial lesions and ovarian endometriomas represent the majority of endometriotic implants within the pelvis, deep infiltrating endometriosis and extra-pelvic endometriosis are the most challenging conditions to face off. On the one hand, severe symptoms of posterior deep infiltrating endometriosis, such as dyschezia, dysuria, dyspareunia and voiding alterations due to neurotrophism and neurotropism, seriously lower quality of life [14]. On the other hand, the definite diagnosis of extra-pelvic endometriosis is difficult and hysteretic and thus maybe delay timely treatment for patients with endometriosis. In addition, some endometriosis lesions may be very small or hidden. Vizzielli et al. tried to use intraoperative near-infrared radiation imaging after intravenous injection of indocyanine green during robotic operations for removing endometriosis lesions to improve detection rates and acquired some beneficial findings [15]. Despite sometimes medical therapy is enough to reduce symptoms and signs [16], in a large number of patients a complete eradication, with nerve-sparing and vascular sparing approach is needed to restore the normal pelvic anatomy and its functions [17].

In view of renal endometriosis is so rare, there are currently no treatment guidelines [23]. Clinical treatment mainly includes symptom relief and radical treatment. Therefore, the treatment of renal endometriosis should be carried out according to the patient’s clinical symptoms, the characteristics of the lesion, and the patient’s reproductive plan [33]. If an asymptomatic patient has no change in the lesions on follow-up review, no definitive renal therapy is usually required [8]. In addition, for patients of reproductive age, hormone therapy, such as oral contraceptives can relieve abdominal pain and is the best treatment option in the short-term [20]. Although renal endometriosis is a benign condition, surgery is usually considered, laparoscopic management in particular, as the feasibility of this treatment has been widely proven to reduce the length of hospital stay [35, 36]. Surgical intervention is required if there is a potential risk of ureteral obstruction and even loss of renal function due to repeated bleeding from endometriosis of the kidneys [37].

In a word, this article reports the clinical features of renal endometriosis of the first case of endometriosis in an ectopic kidney. Although imaging features are helpful in renal endometriosis, the final diagnosis relies on pathological findings.

## Data Availability

The datasets used during the current study are available from the corresponding author on reasonable request.
